# Near-Optimal Foraging in the Pacific Cicada Killer *Sphecius convallis* Patton (Hymenoptera: Crabronidae)

**DOI:** 10.3390/insects3010133

**Published:** 2012-02-10

**Authors:** Joseph R. Coelho, Jon M. Hastings, Charles W. Holliday

**Affiliations:** 1 Institute for Franciscan Environmental Studies, Biology Program, Quincy University, Quincy, IL 62301, USA; 2 Department of Biological Sciences, Northern Kentucky University, Highland Heights, KY 41099, USA; E-Mail: hastings@nku.edu; 3 Department of Biology, Lafayette College, Easton, PA 18042, USA; E-Mail: hollidac@lafayette.edu

**Keywords:** wasp, optimal foraging, cicada, desert, insect, load carriage, flight, force

## Abstract

This study evaluated foraging effectiveness of Pacific cicada killers (*Sphecius convallis*) by comparing observed prey loads to that predicted by an optimality model. Female *S. convallis* preyed exclusively on the cicada *Tibicen parallelus*, resulting in a mean loaded flight muscle ratio (FMR) of 0.187 (N = 46). This value lies just above the marginal level, and only seven wasps (15%) were below 0.179. The low standard error (0.002) suggests that *S. convallis* is the most ideal flying predator so far examined in this respect. Preying on a single species may have allowed stabilizing selection to adjust the morphology of females to a nearly ideal size. That the loaded FMR is slightly above the marginal level may provide a small safety factor for wasps that do not have optimal thorax temperatures or that have to contend with attempted prey theft. Operational FMR was directly related to wasp body mass. Smaller wasps were overloaded in spite of provisioning with smaller cicadas, while larger wasps were underloaded despite provisioning with larger cicadas. Small wasps may have abandoned larger cicadas because of difficulty with carriage.

## 1. Introduction

The effectiveness of a forager can be evaluated by comparing observed outcomes to those expected, based on an optimality model [1,2]. Predaceous solitary wasps have served as useful models for the examination of foraging strategies. One approach to the study of wasps that carry their prey in flight is to compare measured masses of prey actually carried to a predicted optimal prey mass. As yet, no wasp species has been shown to be a perfectly “optimal” forager according to this model [3]. Some species carry less than ideally sized loads, while others carry prey that are larger than expected. Another wasp, *Tachytes chrysopyga obscurus* Cresson, yielded a near-optimal average but a very large variance, indicating little specialization of optimal prey mass [4].

The eastern cicada killer, *Sphecius speciosus* Drury, provides an interesting case. Females emerge, mate, and dig a burrow. After orienting to the burrow entrance, they fly out in search of cicadas with which to mass provision their young. A wasp stings a cicada, resulting in its complete paralysis, and carries the prey in flight back to a nest cell in its burrow, and lays an egg on it [5]. In Illinois and Indiana populations, many females actually carry cicadas (various *Tibicen* spp.) that are larger than that with which they should be able to take off. The wasps compensate for such overloading by climbing trees and descending in flight toward their burrows [6]. However, *S. speciosus* is broadly distributed, ranging from the continental divide in the US eastward through all but the most northeastern states [7]. Thus, it has available a broad range of cicada prey size. In a Florida population that preys nearly exclusively on the diminutive *Neocicada hieroglyphica* Say and *Diceroprocta olympusa* Walker, females are almost always underloaded and appear never to need to climb vegetation or other vertical objects during return provisioning flights [8]. 

The Pacific cicada killer, *S. convallis* Patton, is found through a band of western US states from Washington, Oregon and Idaho south through California and Nevada to Arizona, New Mexico and west Texas [7]. There are essentially no published reports on the biology of this species, though prey records show that *S. convallis* takes cicadas from two species each of *Diceroprocta* and *Tibicen* [9]. We located a large, isolated population of *S. convallis* and postulated that it would make an ideal sister species for comparison to *S. speciosus*, as well as other solitary wasp species whose foraging capacities have been examined. 

For a female cicada killer, carrying the largest cicada possible will make the best use of the limited time and energy she has available. Fortunately, this optimal load size is simple to calculate, as the force production of flying animals is primarily dependent on their flight muscle mass. The maneuverability of a flier is thus dependent on the ratio of flight muscle mass to body mass, or flight muscle ratio (FMR). During foraging, the maximum force produced must at least equal the weight of the wasp plus that of the prey if the wasp is to maintain level flight when laden with prey. At the maximum load mass, the ratio of flight muscle mass to body plus prey mass (or operational flight muscle ratio, FMR_o_) is 0.179 [10]. The goal of the present study is to examine the foraging capacity of *S. convallis* through this model.

## 2. Experimental Section

A very large population of Pacific cicada killers was found nesting on a 3.3-ha field of mine tailings in the ghost town of Ruby (Santa Cruz County), Arizona (31°27'33.18"N, 11°14'02.77"W) in August-September 2009. The mine tailings are the byproduct of approximately 130 years of efforts to extract gold, silver, lead, copper, zinc and tin from the site [11]. The fine, sand-like material provides an excellent substrate for burrowing, although it has a low permeability to water [12]. 

Female Pacific cicada killers loaded with prey were intercepted en route to their burrows, captured with an insect net, immobilized by refrigeration and weighed to the nearest 0.001 g using an Ohaus Adventurer-Pro electronic balance. The body mass of the prey cicada was similarly determined. Right wing length of both wasp and cicada were measured with digital calipers to the nearest 0.01 mm. The head, abdomen, legs and wings of the wasp were removed with scissors and the thorax mass measured. Data are reported as mean ± SEM (N). 

Flight muscle mass of the wasps was estimated as 95% of thorax mass [10] (Marden 1987). Thorax mass was measured in a subsample of 12 cicadas by cutting open the thorax, scraping out the muscle, measuring the mass of the remaining carcass, and subtracting that value from the body mass. Several of the wasps and cicadas were collected as voucher specimens and deposited in the Lafayette College Insect Collection and the Quincy University Life Sciences Museum.

## 3. Results and Discussion

### 3.1. Morphometric Measurements

Female cicada killer right wing length averaged 29.40 ± 0.19(46) mm. Cicada killers weighed 0.991 ± 0.021(46) g and had a thorax mass of 0.418 ± 0.009(46) g resulting in an unladen FMR of 0.401 ± 0.004(46).

**Figure 1:**
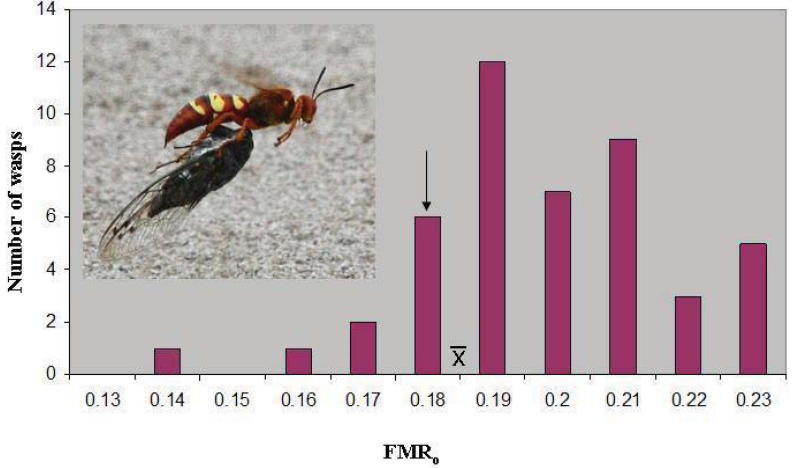
The distribution of operational flight muscle ratios in *Sphecius convallis* foraging on *Tibicen parallelus*. The arrow indicates the marginal flight muscle ratio, where vertical take-off is just possible. 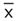 indicates the mean. Inset: Pacific cicada killer female carrying cicada in flight.

All prey of *S. convallis* were identified as *Tibicen parallelus* Davis (Hemiptera: Cicadidae), not only for the present study, but also for the hundreds of specimens examined in the course of other research projects at this site during this period. Cicada right wing length was 36.21 ± 0.20(46) mm. Cicada body mass averaged 1.127 ± 0.024(46) g, resulting in a mean loaded wasp FMR_o_ of 0.187 ± 0.003(46). The latter value lies just above the marginal level, and only seven wasps (15%) were below 0.179 (Figure 1). However, the marginal level lies outside the 95% confidence interval of the mean (0.186–0.189), and the distribution has a skewness of −0.387. The median FMR_o_, 0.186, is essentially identical to the mean.

The nearness of the mean FMR_o_ to the marginal FMR suggests that *S. convallis* is a nearly ideal flying predator with respect to loading. However, the mean loaded FMR is slightly above the marginal level, indicating slight underloading. The marginal FMR is defined as that at which the animal was just able to take off [10]. Therefore, in most load-bearing female Pacific cicada killers a small safety margin exists. That is, wasps are slightly underloaded, which provides them with additional force production during flight beyond that required to lift the cicada and return to the burrow in flight. 

This safety margin may have evolved in response to several selection pressures. Female wasps returning to their burrows with prey appear to have highly variable thorax temperatures [12]. Wasps that do not have optimal thorax temperatures at the time of foraging should experience depressed force production and, hence, decreased ability to carry loads [13]. Slight underloading may thus compensate for decreased load carrying capacity. Another factor potentially driving higher FMR and the resulting greater flight maneuverability is prey theft. We observed theft of prey by kingbirds while the wasps were carrying cicadas. Our observations suggest that theft pressure can be intense at times [12]. Therefore, laden wasps with greater FMR would benefit from an improved ability to evade such thievery. Although female cicada killers with prey did not always land at the burrow entrance, they typically landed at least within a meter or two and reached the entrance by walking overland. We never observed wasps climbing objects with their prey, and, in any case, many areas of the mine tailings had no vertical objects available for climbing. Our data indicate that there would seldom be a need to do so, as the wasps have sufficient capacity to fly with their prey.

### 3.2. Effect of Wasp Size on Cicada Body Mass

The cicada was heavier than the wasp in 35 of 46 wasp/cicada pairs, and on average prey cicadas were significantly heavier than wasps by body mass (P < 0.00005, T-test). A nearly even sex ratio among the prey was observed, as 22 of 46 cicadas were males. Neither wasp body mass, thorax mass, nor right wing length (all indices of size) was significantly related to cicada body mass by linear regression (R^2^ < 0.05, N = 46). However, it appeared that small wasps used the smallest prey, while larger wasps carried larger cicadas. Hence, wasp data were divided into two groups: large wasps, those above the median thorax mass (0.4235 g), and small wasps, those below the median. Thorax mass was chosen as the best index of body size because body mass may vary with nutritional status, hydration state, and number of eggs laid, while wing length is influenced by wear. Large wasps had thorax mass of 0.463 ± 0.006g and carried cicadas weighing 1.181 ± 0.033 g. Small wasps had thorax mass of 0.372 ± 0.009 g and carried cicadas weighing 1.073 ± 0.032 g. Prey mass was significantly greater in large wasps as compared to small wasps (P = 0.011, T-test). 

The size of available cicada prey is a good predictor of the sizes of male and female *S. speciosus*, though it is not clear whether this effect is nutritional or evolved [14]. *S. speciosus* preys on at least 30 species from five genera of cicadas which range a great deal in size [9]. This large prey diversity may have led to the greater measured variation in wasp size. The coefficient of variation of wing size in female *S. speciosus* is 10.1% in a Newberry, FL, population and 8.2% in an Easton, PA, population. The coefficient of variation of wing length was only 4.3% in female *S. convallis* and 3.8% in *T. parallelus* in the present study, suggesting that there is a tight relationship between the Ruby, AZ, predator and prey populations. Thus, it seems likely that the consistency in *T. parallelus* size has led to low variation in *S. convallis* size. The fact that this population of cicada killers preys on adult cicadas of a single species with low size variation may have allowed stabilizing selection to adjust the size of adult female wasps to a nearly optimal value. 

If cicada killers have a 24.6% conversion efficiency [12] of cicadas into wasp mass, then four cicadas of average mass will yield one 1.11-g female wasp, which is approximately the average observed mass at Ruby, AZ. A female of that mass is never overloaded. Consistency in provisioning of cells, therefore, is critical to the production of females large enough to carry the prey. Underprovisioning a female larva would result in an undersized female adult that would, in turn, be a poor provisioner. Selection must act on provisioning behavior to maintain the size of adult females.

### 3.3. Effect of Wasp Body Mass on FMR_o_

**Figure 2:**
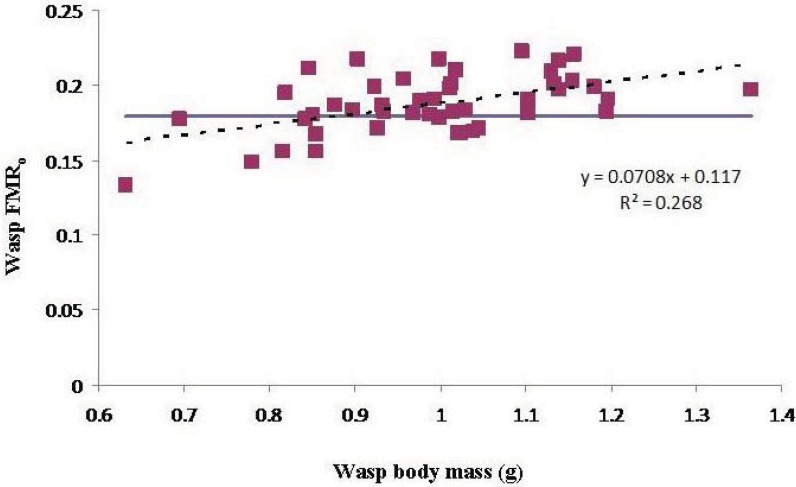
The effect of wasp body mass on operational flight muscle ratio. The slope of the regression line (dashed) is significant (P < 0.01). The blue line indicates the marginal flight muscle ratio.

There was a significant effect of wasp M_b_ on FMR_o_, indicating that smaller wasps were overloaded more often than larger wasps (Figure 2).

Flight muscle mass of a subsample of cicadas was 0.255 ± 0.014 (12) g, resulting in FMR of 0.225 ± 0.012 (12) g. As the FMR of female wasps is 60% greater than that of the cicadas, the wasps should have much greater maneuverability and, hence, no difficulty capturing cicadas on the wing. A similar relationship was demonstrated between *S. speciosus* and various *Tibicen* prey species [6], although we are not aware of any confirmed accounts of *Sphecius* attacking cicadas in flight.

Some studies appear to indicate that cicada killers do not make choices based on cicada species during foraging. Generally, the species taken are often reflective of those available. Cicada killers may have a generalized cicada search image, and are opportunistic in what they will take. Occasionally, eastern cicada killers even take periodical cicadas [15], species which they might encounter only every 13 or 17 years, and even then rarely, as the early summer season of *Magicicada* overlaps little with the late summer season of cicada killers. 

On the other hand, we never observed *S. convallis* provisioning with The Grand Western Flood Plain Cicada, *Tibicen cultriformis* (Davis), which was occasionally heard singing in the area of the *S. convallis* colony at Ruby, AZ. Its rarity at the site and large size may have resulted in its unsuitability as prey. We heard only one *T. cultriformis* singing at a time, suggesting that their numbers were few; though we were unable to collect any in the immediate vicinity, we collected several from lower altitudes, where they were much more abundant, and made morphometric measurements. The mean body mass was 3.246 ± 0.083 (14) g. We never observed *S. convallis* foraging on *T. cultriformis*. Indeed, if the average *S. convallis* attempted to carry the average *T. cultriformis*, a FMR_o_ of 0.097 would result and vertical take-off would be impossible. Hence, even if *S. convallis* attempted to provision with *T. cultriformis*, we would be very unlikely to collect them at nest sites. As a result, it is unclear whether *T. cultriformis* plays a role in *S. convallis* foraging ecology.

There was some evidence that suggested the possibility that *S. convallis* females made size-based choices among individuals of *T. parallelus*. In spite of taking generally smaller cicadas, small wasps were the most overloaded. This pattern might be explained, not by prey choice, but by unsuccessful provisioning attempts by small, overloaded wasps. Several lines of evidence suggest indirectly that this occurs. In Figure 1, the slight negative skewness indicates that the bulk of the values were higher than the mean. In other words, overloaded wasps (those with low FMR_o_) were underrepresented in the distribution. If a female takes a cicada that is too large, then it is more likely to be abandoned on the flight back to the burrow; the foraging bout is a failure, and the female goes on to hunt for more cicadas. Consequently, we often find paralyzed cicadas under trees in areas where female eastern cicada killers are hunting [12], though we did not observe this phenomenon directly in *S. convallis*. It also seems more likely that overloaded wasps, having less maneuverability, would be successfully attacked by thieving birds. Failure of small wasps to provision with large cicadas could have produced the statistical result observed; it cannot be concluded that large wasps took cicadas that were larger than expected. Availability of prey was apparently low during the present study, as few cicadas were heard calling in the study area, and foraging bouts required ~8 h/cicada [12] *vs.* 24 min/cicada for *S. speciosus* in Pennsylvania [16]. It is not surprising, then, that clear evidence for prey choice was not detectable.

## 4. Conclusions

A recent review [17] shows that optimality models frequently fail when applied to predators of mobile prey, in part owing to variation in capture success. Cicadas generally rely on crypsis rather than flight for defense, and have little in the way of postcapture defense, except perhaps vigorous buzzing. Cicada killers have much greater flight maneuverability than cicadas, and should have no difficulty in capturing them if they should take flight. Hence, cicadas are essentially immobile prey, improving the likelihood that cicada killer foraging will support an optimality model.

Indeed, evidence for size-based prey choice in cicada killers is mounting. *Sphecius grandis* in the Chiricahua Mountains of Arizona provisioned with *T. duryi* and *T. parallela*, but never provisioned with the much smaller *T. chiricahua* or *Platypedia putnami*. However, these latter two species were active early in the summer, and few were still alive during the active period of the female wasps [12,18]. More recently, a population of variably sized *S. speciosus* with access to a variety of cicada sizes was examined [8]. While small wasps took smaller cicadas, as expected, large wasps also took large cicadas, in a significantly greater proportion than should be expected. 
